# Viral Vectors for Plant Genome Engineering

**DOI:** 10.3389/fpls.2017.00539

**Published:** 2017-04-11

**Authors:** Syed Shan-e-Ali Zaidi, Shahid Mansoor

**Affiliations:** Molecular Virology and Gene Silencing Laboratory, Agricultural Biotechnology Division, National Institute for Biotechnology and Genetic EngineeringFaisalabad, Pakistan

**Keywords:** CRISPR/Cas9, DNA replicon, geminivirus, genome engineering, *Tobacco rattle virus* (TRV)

## Abstract

Recent advances in genome engineering (GE) has made it possible to precisely alter DNA sequences in plant cells, providing specifically engineered plants with traits of interest. Gene targeting efficiency depends on the delivery-method of both sequence-specific nucleases and repair templates, to plant cells. Typically, this is achieved using *Agrobacterium* mediated transformation or particle bombardment, both of which transform only a subset of cells in treated tissues. The alternate *in planta* approaches, stably integrating nuclease-encoding cassettes and repair templates into the plant genome, are time consuming, expensive and require extra regulations. More efficient GE reagents delivery methods are clearly needed if GE is to become routine, especially in economically important crops that are difficult to transform. Recently, autonomously replicating virus-based vectors have been demonstrated as efficient means of delivering GE reagents in plants. Both DNA viruses (*Bean yellow dwarf virus, Wheat dwarf virus* and *Cabbage leaf curl virus*) and RNA virus (*Tobacco rattle virus*) have demonstrated efficient gene targeting frequencies in model plants (*Nicotiana benthamiana*) and crops (potato, tomato, rice, and wheat). Here we discuss the recent advances using viral vectors for plant genome engineering, the current limitations and future directions.

## Introduction

Genome engineering (GE) refers to the strategies and techniques developed for the targeted, specific modification of the genetic information of living organisms. GE technologies have recently evolved as promising tools for improvement of a wide range of organisms, including plants (Schaeffer and Nakata, 2016). The major advantage of GE is that it enables a specific sequence on a chromosome be modified, thereby increasing the precision of the gene disruption, correction or insertion, offering perfect reproducibility (Songstad et al., 2017). One of the primary challenges in engineering plant genome is the choice of vectors that are modified in a systematic manner to deliver reagents for GE.

For a long time, plant viruses have been used as vectors for several purposes including the commercial production of useful proteins (Rybicki, 2009). The efficient machinery and comprehensive genome structure makes viral genomes excellent choice to be used as vectors. Autonomously replicating virus-based vectors provide alternative means to deliver GE reagents to plant cells. Among these are the RNA viruses, which for monocots include *Wheat streak mosaic virus* (WSMV) and *Barley stripe mosaic virus* (BSMV) (Lee et al., 2012) and *Tobacco rattle virus* (TRV) for dicots. Single-stranded (ss) DNA viruses, as geminiviruses, have been also widely adopted as vectors for diverse crops. These viruses can be modified to carry heterologous coding

sequences, and protein expression has been achieved in important crops like wheat, barley, corn, oat, and rye (Choi et al., 2000). Recent development in GE technologies have urged scientists to incorporate viral vectors and utilize them for the efficient delivery of GE reagents in plant cell (**Table 1**).

**Table 1:** Important viral vectors for plant genome engineering.

Virus type	Virus vector	GE platform	Plant species	Target	Reference
DNA virus	BeYDV	CRISPR and TALEN	*Solanum lycopersicum*	*ANT1*	Cermak et al., 2015
	BeYDV	ZFN, TALEN and CRISPR	*Nicotiana tabacum*	P-*GUS:NPTII*	Baltes et al., 2014
	BeYDV	CRISPR	*Solanum tuberosum*	*stALS1, stALS2*	Butler et al., 2015
	BeYDV	CRISPR and TALEN	*Solanum tuberosum*	*ALS1*	Butler et al., 2016
	CaLCuV	CRISPR	*Nicotiana benthamiana*	*PDS*	Yin et al., 2015
	WDV	CRISPR	*Triticum aestivum*	*Ubi, MLO, GFP*	Gil-Humanes et al., 2017
	WDV	CRISPR	*Oryza sativa*	*GFP, GUS*	Wang et al., 2017

RNA virus	TRV	ZFN	*Nicotiana tabacum* and *Petunia hybrida*	*uidA*	Marton et al., 2010
	TRV	Meganuclease	*Nicotiana alata*	*DFR*	Honig et al., 2015
	TRV	CRISPR	*Nicotiana benthamiana*	*PDS*	Ali et al., 2015b
	TRV	CRISPR	*Nicotiana benthamiana*	*PDS, PCNA*	Ali et al., 2015a
	TRV	CRISPR	*Nicotiana benthamiana*	Plant virus	Ali et al., 2015c
	TRV	CRISPR	*Nicotiana benthamiana*	Plant virus	Ali et al., 2016

*Acetolactate synthase1 (ALS1), Solanum tuberosum acetolactate synthase1 (StALS1), green fluorescent protein (GFP), β-Glucuronidase [GUS] reporter controlling gene (uidA), Promoter of GUS and neomycin phosphotransferase (P-GUS:NPTII), Bean yellow dwarf virus (BeYDV), Cabbage leaf curl virus (CaLCuV), zinc finger nucleases (ZFNs), transcription activator like effector nucleases (TALEN), clustered regularly interspaced short palindromic repeats (CRISPR), Tobacco rattle virus (TRV), genome engineering (GE), phytoene desaturase gene (PDS), proliferating cell nuclear antigen (PCNA), ubiquitin gene (Ubi), MildewLocusO (MLO), dihydroflavonol 4- reductase (DFR).*

## Genome Engineering Platforms

There are four major GE platforms based on the use of: (1) meganucleases, (2) zinc finger nucleases (ZFNs), (3) transcription activator like effector nucleases (TALEN) and (4) clustered regularly interspaced short palindromic repeats (CRISPR)/CRISPR associated9 (CRISPR/Cas9). All these platforms share common feature of utilizing sequence-specific nucleases (SSNs), hence referred to as ‘designer nucleases.’ The fate of double stranded breaks (DSBs) introduced by the SSNs is either non-homologous end joining (NHEJ) or homology-directed repair (HDR) (Qi et al., 2016). NHEJ occurs when cellular repair machinery force joins these DSBs and doing so an insertion or deletion (indel) of few nucleotides takes place. In most cases the user specific sequence is the coding sequence of a specific protein, and indel formation at target site causes an early stop codon, forming a truncated, usually non-functional, version of the target protein, whereas HDR uses longer stretches of sequence homology to repair DNA lesions (Wright et al., 2016).

ZFNs and TALENs have been used for targeted editing of plant genomes (Voytas, 2013). However, the customization of ZFNs and TALENs requires protein engineering for each user-selected targeted, a resource intensive and time-consuming process. Recently, bacterial and archaeal natural immunity system that targets and destroys invading nucleic acids has been adapted for GE across eukaryotic species. The CRISPR/Cas9 system has been used in diverse plant species such as rice, wheat, maize, tomato, potato, *Nicotiana benthamiana* and *Arabidopsis thaliana* for targeted genome editing (Noman et al., 2016). The CRISPR/Cas9 system is comprised of the Cas9 endonuclease of *Streptococcus pyogenes* and a synthetic guide RNA (gRNA), which combines functions of CRISPR RNA (cRNA) and *trans*-activating cRNA (tracrRNA) to direct the Cas9 protein to the DNA target sequence preceding the protospacer-associated motif (PAM) (NGG in the case of *S. pyogenes*) (Cong et al., 2013). Because the specificity of the system is determined by the 20-nucleotide sequence of the gRNA, it allows for unprecedented and facile GE. Further, the CRISPR/Cas9 system can be used to simultaneously edit multiple genomic targets (Cong et al., 2013).

## Geminiviruses As Vectors For Genome Engineering

Geminiviruses (family *Geminiviridae*) (Briddon, 2015) are widespread around the globe and have the ability to infect a wide variety of plant species like wheat, maize, cotton, tomato, cucurbits, beans, legumes, fruits, ornamental plants, and common weeds (Nawaz-Ul-Rehman and Fauquet, 2009; Rey et al., 2012). Geminiviruses have a small genome of ∼2.8 Kb containing four to six overlapping open reading frames (ORFs); both in the sense and complementary sense orientation. They are transmitted via insect vectors like whitefly *Bemisia tabaci* and leaf hoppers.

**FIGURE 1:**
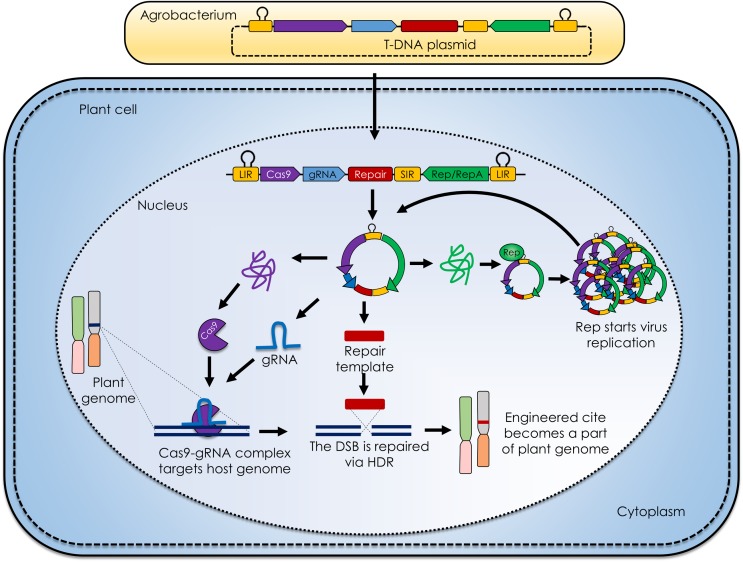
**Geminivirus mediated plant genome engineering.** The generalized molecular mechanism of geminivirus based replicons for delivering clustered regularly interspaced short palindromic repeats (CRISPR) /CRISPR associated9 (CRISPR/Cas9) reagents in plant cell. The deconstructed virus genome is cloned in T-DNA, transformed in *Agrobacterium tumefaciens* and delivered to plants, namely agroinfiltration. The DNA is inserted into the plant cell nucleus where the single stranded viral DNA is converted into the double stranded DNA replicative form and RNA is transcribed. The engineered viral transcript contains two long intergenic regions (LIRs) spanning the Cas9, guide RNA (gRNA), repair template, short intergenic region (SIR) and a replication associated protein (Rep). The Cas9 and gRNA combine to from a Cas9-gRNA complex that recognizes and binds to the target site adjacent to the protospacer adjacent motif (PAM), where Cas9 nuclease produces a double stranded break (DSB). In case of homolog directed repair (HDR), the DSB is repaired with the aid of a repair template that becomes the part of plant genome. Rep protein on the other hand starts the replication of viral DNA producing numerous transcripts that further follow the same pipeline and ensure efficient CRISPR/Cas9 mediated plant genome engineering.

Certain features of geminiviruses make them outstanding for plant GE: (1) geminiviruses are able to infect a wide range of host plant species from various families, making these efficient vectors for multiple hosts at once; (2) require only one protein, Rep (replication associated protein; RepA in case of mastreviruses), to initiate replication inside the host cell, and it can be expressed under its natural promoter within intergenic region or other user specified constitutive/inducible promoters (**Figure 1**) (Baltes et al., 2014); (3) replicate inside host cells via homologous recombination-dependent replication, in addition to rolling circle replication, reverting host cells to S phase, suitable for homologous recombination if supplemented with SSN and complementary target sequences (Richter et al., 2016) (**Figure 1**) and (4) replicate efficiently inside host cell and produce high amounts of replicons, in turn producing a lot of SSNs and target sequence if used as vector for GE, considerably enhancing the targeting efficiency (Hanley-Bowdoin et al., 2013).

Geminiviruses have been engineered as vectors for the expression of heterologous proteins in plants (Lozano-Duran, 2016). Whereas the cargo capacity of these viruses is quite restricted, they can be converted into non-infectious replicons by replacing genes important for infection and cell-to-cell movement with heterologous sequences, such as SSN expression cassettes and repair templates. To achieve this goal, movement protein (MP) and coat protein (CP) coding sequences of geminiviruses have been removed (**Figure 1**), thereby eliminating the possibility of cell-to-cell movement as well as plant-to-plant insect-mediated transmission. The lack of the CP increases the copy number of dsDNA replicon intermediates, likely because CP is not available to sequester and package ssDNA into virions, and loss of CP/Rep interactions represses viral replication.

Four studies stand out on the use of geminivirus vectors for GE (Baltes et al., 2014; Cermak et al., 2015; Gil-Humanes et al., 2017; Wang et al., 2017) (**Table 1**). Baltes et al. (2014) developed a deconstructed version of *Bean yellow dwarf virus* (BeYDV) and used it to efficiently deliver ZFNs and a repair template to tobacco cells to achieve gene targeting (GT) at an integrated reporter gene. Efficient replication and accumulation of geminivirus based replicons for transient expression of SSNs was demonstrated with efficient HDR thereafter. Furthermore, the BeYDV replicons have shown considerable cargo capacity and could deliver TALENs and CRISPR/Cas9 reagents (Baltes et al., 2014). Cermak et al. (2015) used BeYDV based replicons for GT and insertion of a strong promoter upstream of a tomato gene that regulates anthocyanin synthesis. GT frequencies were ∼12-fold higher than what could be achieved using standard *Agrobacterium* T-DNA delivery (Cermak et al., 2015). Gil-Humanes et al. (2017) developed replicons based on a *Wheat dwarf virus* (WDV) for precise genome editing of cereal crops. The WDV-derived replicons amplified and expressed heterologous proteins in wheat, corn, and rice. The replication and protein expression of the WDV system was also characterized in wheat cells, and compared to different replicon architectures to optimize WDV as a vector for delivering CRISPR/Cas reagents and donor templates. WDV replicons increased GT efficiency greater than 10-fold in wheat cells, and they were also able to promote multiplexed GT, achieving within the same cell targeted integration of different reporter genes in different loci of the polyploid wheat genome (Gil-Humanes et al., 2017). Recently, Wang et al. (2017) have shown CRISPR/Cas9 mediated GT and efficient HDR (as high as 19.4%) in rice, using a WDV-based replicon system. Two studies have used similar approach for the targeted GE in potato (Butler et al., 2015, 2016). These studies overcame three important barriers: (1) increasing the efficiency of HDR in plants; (2) using geminivirus vectors for GE in plants and (3) development of permanent transgenic lines using geminivirus mediated HDR.

Another novel use of geminivirus vectors has been developed by Yin et al. (2015) the “virus based gRNA delivery system for CRISPR/Cas9 mediated plant genome editing (VIGE).” VIGE makes use of Cas9 over expression in plants (*N. benthamiana* so far) and transient delivery of geminivirus vectors with sgRNA targeting the gene of interest. This system can be used for generation of knock-out libraries, as an alternative to the virus induced gene silencing (VIGS) (Yin et al., 2015). All the above mentioned studies are exciting and promising, however, certain limitations needed to be addressed (discussed in Section “Conclusion and Future Prospects”).

Begomoviruses (genus *Begomovirus* in the family *Geminiviridae*) are frequently associated with DNA satellite molecules including betasatellites, alphasatellites, and deltasatellites (Briddon and Stanley, 2006; Zhou, 2013; Lozano et al., 2016). Betasatellites are interesting in several aspects since a single species of betasatellite has the ability to be transreplicated by diverse helper begomoviruses and thus can infect a wide range of host plants. Betasatellites are half the size of helper viruses (∼1.4 kb) and have a single gene in complementary sense orientation that encodes for beta-C1 protein, a pathogenicity determinant. This single ORF of a well characterized Cotton leaf curl Multan betasatellite (CLCuMB) has been removed to utilized as a vector with several helper viruses. CLCuMB has been used as a delivery vector for the production of foreign protein *Bcl-2* in plants (Kharazmi et al., 2016). The potential of betasatellites as a vector for GE reagent remains to be explored.

CRISPR/Cas9 technology has also been used to engineer resistance against geminiviruses (Ali et al., 2015c, 2016; Baltes et al., 2015; Ji et al., 2015) and potyviruses (Chandrasekaran et al., 2016; Pyott et al., 2016) either by directly targeting and cleaving virus genome (Ali et al., 2015c, 2016; Baltes et al., 2015; Ji et al., 2015) or by altering plant genome to trigger immunity against invading viruses (Chandrasekaran et al., 2016; Pyott et al., 2016). This technology has been extensively reviewed and readers are directed to the following detailed review (Zaidi et al., 2016b) and research highlights (Chaparro-Garcia et al., 2015; Zlotorynski, 2015; Zaidi et al., 2016a) for further details on this topic.

## *Tobacco rattle virus* As Vector For Genome Engineering

*Tobacco rattle virus* (genus *Tobravirus*, family *Virgaviridae*) is a positive single stranded RNA (+ssRNA) pathogenic plant virus that infects over 400 plant species from 50 families. It is naturally transmitted by nematodes of the family *Trichodoridae*; and can also be mechanically and seed transmitted. TRV has two genome components, TRV1 (or RNA1) and TRV2 (or RNA2). TRV1 is essential for viral movement and contain genes encoding 134- and 194-kDa replicase proteins, a 29-kDa MP and a 16-kDa cysteine-rich protein whose function is not fully known. The TRV2 genome varies among different isolates of this virus and has genes encoding the CP and non-structural proteins. These non-structural proteins are implicated in nematode transmission, but they are not essential for experimental infection. Therefore, for use as a vector, the two non-structural protein–encoding genes in TRV2 can be replaced with multiple cloning sites for inserting fragments of interest (Senthil-Kumar and Mysore, 2014).

TRV as a vector meets several important requirements for highly efficient and multiplexed editing: (1) can systematically infect a large number of plant species; (2) the virus is easily introduced into plants via *Agrobacterium* and delivery into growing points of the plant; (3) the small genome size facilitates cloning, multiplexing, library constructions, and agroinfections; and (4) the virus RNA genome does not integrate into plant genomes. TRV is an efficient vector for VIGS, facilitating functional genomics in diverse plant species.

TRV has shown to be promising as a vector for GE (**Table 1**). A non-transgenic approach was adapted for ZFN delivery and production of mutant plants using TRV-based expression system for indirect transient delivery of ZFNs into a variety of tissues and cells of intact plants (Marton et al., 2010). For TRV to have the most utility as a vector for GE, it would be desirable if the virus infected germline cells, making it possible to harvest mutant seed from infected plants. A TRV vector expressing a site-specific meganuclease was developed by (Honig et al., 2015) who demonstrated efficient and heritable mutations in dihydroflavonol 4- reductase (DFRa), a NADPH-dependent enzyme that converts dihydroflavonols to their corresponding leucoanthocyanidins and its inactivation causes a visible phenotype of reduced purple pigmentation in anthocyanin-accumulating organs, such as flower petals. The mutations were heritable in the M1 progeny, and some of these were inherited by at least two further generations. Ali et al. (2015a) developed a TRV-mediated gRNA delivery system that bypasses the requirement for transformation and/or regeneration of each user-defined target sequence, amenable to multiplexing, and in which editing efficiencies and applicability across plant species would be significantly improved. To construct this virus-mediated genome editing system, these authors generated Cas9-overexpressing (Cas9-OE) *N. benthamiana* transgenic lines, and then used *Agrobacterium* to deliver an optimized TRV for gRNA delivery. This opened up new possibilities to produce plants with desired traits using CRISPR/Cas9 without the involvement of laborious and time intensive tissue culture practices.

## Conclusion And Future Prospects

Genome editing is a promising tool for introduction of novel traits, but its application is limited in plants because of inefficient HDR. The bottleneck of utilizing genome editing for plant improvement is that the primary DNA repair mechanism in plants is NHEJ, and a lot of efforts are underway to improve the efficiency of HDR in plants. The efficient and high production of SSN reagents via geminivirus vectors can be a potential solution to this problem. Indeed, geminivirus vectors have recently been developed as promising tools to improve HDR in plants. However, there are still several issues needed to be addressed for improvement of this recent technique. Regenerating plants transformed with geminivirus vectors has proven extremely difficult, mainly because geminivirus proteins interact with several cellular proteins to facilitate viral replication but in turn compromising the integrity of host cell. A possible solution to this would be the optimization of strictly inducible geminivirus vectors, e.g., that expression start only when the plant reaches a certain biomass. In transient systems, the geminivirus vectors are limited to infiltrated leaves only, and the cell-to-cell movement is restricted because of the unavailability of movement related proteins. This greatly limits the levels of expressed SSNs, but these proteins should necessarily be removed to facilitate the strict genome size limitation of geminiviruses. A plausible solution could be to engineer betasatellites for delivery of GE reagents or separately expressing the MP/s under another vector or using bipartite begomovirus vectors which have movement related proteins on a separate genomic component. The TRV mediated CRISPR/Cas9 and the VIGE systems are currently limited to the availability of the transgenic lines (of any plant species under experimentation) stably expressing Cas9. As more and more crop species are being engineered with the CRISPR/Cas9 system, Cas9 overexpression seeds are being available for wider range of species. Nevertheless, for the successful application of these technologies, this limitation must be addressed, especially for the promising technologies like TRV-mediated heritable GE.

Recently developed TRV mediated CRISPR/Cas9 delivery system has the potential to bypass the laborious and time consuming tissue culture practices to develop plants with desirable engineered traits. TRV have been used with *Agrobacterium* to efficiently deliver ZFNs and CRISPR/Cas9 reagents for genetic modification in plants and the use of heterozygous Cas9 overexpressing plants with this facile genome-editing platform has allowed the engineering and production of plants free of foreign DNA. This might overcome the regulatory hurdles that impede the commercialization of engineered plants. However, the limitations in carrying capacity of TRV and other RNA viruses prevent their use beyond expression of relatively small SSNs and sgRNAs and are unable to efficiently deliver DNA repair templates. Geminiviruses may be able to overcome the limitations of RNA viruses by allowing a larger carrying capacity and producing a DNA replicon capable of acting as a repair template for GT. Moreover, the geminivirus associated satellites, like betasatellites, have the potential to act as efficient delivery vectors. However, the utilization of betasatellites as vectors for GE is yet untried. Furthermore, the efficiency of a combined delivery system, utilizing both geminiviruses and TRV, for delivery of different reagents, also remains to be explored.

## Author Contributions

SZ and SM provided the outlines of the review and contributed the key ideas. SZ wrote the manuscript and prepared the figure. SM worked on and improved the original draft and figures.

## Conflict of Interest Statement

The authors declare that the research was conducted in the absence of any commercial or financial relationships that could be construed as a potential conflict of interest.
